# Benefits and risks of antipsychotic discontinuation in people with first and multi-episode psychotic disorders or with schizophrenia: why, when, how and in whom?

**DOI:** 10.1038/s41537-025-00700-3

**Published:** 2025-12-17

**Authors:** Christoph U. Correll, Jose M. Rubio, John M. Kane

**Affiliations:** 1 Charité—Universitätsmedizin Berlin, Department of Child and Adolescent Psychiatry, Berlin, Germany; 2 German Center for Mental Health (DZPG), partner site Berlin, Berlin, Germany; 3 German Center for Child and Adolescent Health (DZKJ), partner site Berlin, Berlin, Germany; 4 Einstein Center for Population Diversity (ECPD), Berlin, Germany; 5 Northwell, New Hyde Park, NY USA; 6 Donald and Barbara Zucker School of Medicine at Hofstra/Northwell, Department of Psychiatry and Molecular Medicine, Hempstead, NY USA; 7 The Feinstein Institute for Medical Research, Center for Psychiatric Neuroscience, Northwell Health, New Hyde Park, NY USA

**Keywords:** Human behaviour, Drug delivery

Most treatment guidelines for schizophrenia recommend long-term antipsychotic use for relapse prevention^1^. However, the reality is that most people living with this chronic condition discontinue treatment recurrently over time^2^. In “Helping people to discontinue antipsychotics: if, when and how” Drs Moncrieff and Horowitz provide recommendations to clinicians about when and how to support patients through the process of antipsychotic treatment discontinuation.

The fact that most individuals on antipsychotics often discontinue treatment cannot be ignored, and stakeholders need to address this important issue. Frequent treatment discontinuation cannot be conflated with antipsychotics being generally unnecessary or discontinuation being often in the best interest of the user. In their manuscript, Moncrieff and Horowitz take the implicit position that for most of those individuals with psychotic conditions, without consideration if this is also true for people fulfilling criteria for schizophrenia, who express interest in discontinuing antipsychotic treatment the medical advice should be to recommend and facilitate such discontinuation. We argue that aligning with patient-focused and recovery-oriented care, this is often not true. Our basic arguments are that at least for people diagnosed with schizophrenia, as opposed to the very heterogeneous samples of people with first-episode psychosis, which should not be confused in these arguments: (1) A balanced and comprehensive review of the literature shows that at the group-level antipsychotic discontinuation or no antipsychotic treatment is most often associated with negative outcomes, in some cases life-threatening and irreversible, with evidence of premature mortality and limited evidence of advantages^3–5^, (2) As of today, there are no validated methods to predict whether discontinuation will be favorable for any given individual, hence the recommendation to interrupt treatment is done with inadequate consideration of the potential consequences. More than just following patient preference, recovery-oriented care is based on establishing a collaborative partnership, empowering patients to make informed decisions balancing autonomy with clinical expertise and ethical responsibilities. Our view is that recommending widespread antipsychotic discontinuation goes against the principles of recovery-oriented care.

The authors make sweeping and out of context statements intended to bolster their arguments that are not readily supported. For example, the authors write: “since there is no question that antipsychotics cause a high burden of debilitating and harmful adverse effects.” Like any other medications, antipsychotics may cause side effects, with different profiles among the broad range of options. The frequency, severity, duration of adverse effects and their impact on well-being and functioning need to be considered along with potential benefits and potential consequences of alternatives, including treatment discontinuation. For instance, the “debilitating and harmful” consequences of relapse following treatment discontinuation may be far larger than what is being experienced with a given antipsychotic, or what would be expected from an alternative drug with a better tolerability profile.

In another example, the authors write ‘there is little evidence that antipsychotics target a pathological mechanism’, which is simply not true. The pathophysiology of psychosis is obviously complex and likely heterogenous, not attributable to a single mechanism. However, there are decades worth of data showing that dopaminergic dysregulation in the striatum plays a causal role in the emergence of psychotic symptoms^6^. There is indeed evidence that antipsychotic drugs engage such pathophysiology, with improvements in striatal functional connectivity being correlated with antipsychotic benefits^7^.

In yet another example, the authors write: “not everyone who stops antipsychotics relapses”. About 10–20% of individuals with schizophrenia may have only one episode^8^, with higher rates in people with the heterogeneous condition of first episode psychosis, but this is not a reason to recommend indiscriminate antipsychotic discontinuation, especially for people suffering from schizophrenia. Notably, these high rates of relapse after antipsychotic discontinuation extend to patients with first-episode schizophrenia-spectrum disorders stabilized for 2–3 years on fluphenazine decanoate where the antipsychotic dose reduction occurred over the 6 months, and was thereafter inherently very slow and where the 2-year relapse rate was 97%^9^. To date, there is no validated method with near acceptable accuracy (e.g., accuracy >80%) to predict whether someone diagnosed with schizophrenia will be in the lucky 3–20% who do not relapse after discontinuation.

Relapse not only poses potential danger to self/others, but may also be associated with lower efficacy of antipsychotics when reintroduced. In one of the clearest examples, early phase individuals in remission were randomly allocated to antipsychotic discontinuation vs maintenance treatment for 1 year and then returned to naturalistic treatment and then followed up at year 10 after study entry. At that point, the dosages of antipsychotics were comparable between the 2 groups, yet those who had discontinued treatment had significantly poorer clinical outcomes (risk ratio 1.84, *p* = 0.01)^10^. Similarly, other studies have shown that reintroduction of antipsychotics after relapse following treatment discontinuation is associated with lower treatment response than in the past for the same individual^11^, or that the dosages used after each rehospitalization for psychosis relapse increases progressively over time without evidence of greater benefit and less benefit at regular doses^12^.

The authors also mention that the positive effects of antipsychotics are exaggerated while the evidence for discontinuation of maintenance treatment is ‘mixed’. We again believe that a balanced and critical appraisal of the evidence disagrees with these statements^3–5^. The authors write ‘the protective effects of antipsychotics may have been exaggerated due to the possibility that withdrawal itself is associated with adverse effects, including relapse’. While ‘rebound psychosis’ is a theoretical possibility, the evidence for its existence is rather limited. For instance, studies interrupting antipsychotics abruptly have not found greater relapse rates than studies with more progressive discontinuation^13^ and even after 6 months of long-acting injectable dose reduction 97% of people 2–3 years stabilized after their first episode schizophrenia relapsed over 2 years^9^. Furthermore, studies on the risk of relapse associated with speed of discontinuation vs. D2 occupancy levels by antipsychotics show that the latter is a statistically significant predictor, rather than the former^14^. Additionally, we would argue that rather than ‘mixed’, the preponderance of the evidence is against the advantages of treatment discontinuation. The RADAR study notoriously found that discontinuation was not associated with better functioning or improvement on side effects, while the risk of relapse was much greater for those who discontinued antipsychotics^15^. Similarly, the MEFISOS study showed an advantage of treatment maintenance during the first 2 years during the controlled phase, while the results of the 7-year timepoint were confounded by the high attrition of individuals with schizophrenia (vs other types of psychotic disorders) and lack of controlled interventions between the 2 groups (e.g., at year 7 the actual difference of antipsychotic exposure between the 2 original groups of the controlled phase was of 1 mg of haloperidol equivalents). Similarly, the GARMED study was underpowered, and dose reduction was not fixed, but rather titrated based on symptom severity, which limits conclusions.

Continuity of treatment is an issue, not only with antipsychotics, but also with other drugs that are used long-term, like oral antidiabetics, anti-asthma medications, or antihypertensives. Antipsychotics, however, carry additional challenges, related to the stigma still associated with psychotic disorders, which make daily pill taking a reminder of the illness, cognitive symptoms, and in some cases limited insight into the illness. Prescribers need to proactively address these barriers within a recovery-oriented and patient-centered framework, which may mean in some cases to make medical recommendations that may not necessarily align with the initial expressed interest of the client. The definition of capacity to make a decision about treatment discontinuation when at the group level there is high risk of relapse is insufficiently described in the authors’ manuscript, yet we believe to be critical to this issue. Often, prescribers may find themselves in a conflict between client independence and medical recommendations and ethical obligation to not do harm which needs to be recognized, discussed with the relevant stakeholders, and resolved on a case-by-case basis. In other situations, antipsychotic discontinuation may be driven by a poor efficacy:side effect balance. Again, the question is what are the likely benefits, disadvantages and alternatives available, rather than choosing discontinuation before carefully addressing these issues. Often, approaching symptom remission may make the medication seem unnecessary, especially if there is perception of side effects without immediate benefit, as relapse prevention would be a gain only in the future. Therefore, patients should be reminded that in addition to the acute treatment of psychosis, antipsychotic drugs are used for relapse prevention, as a prophylactic measure, akin to vaccines preventing infections. The evidence shows that most individuals who discontinue treatment return to it^2^, but now possibly with lower efficacy and after possibly experiencing irreversible biopsychosocial consequences.

In conclusion, until we have validated and sufficiently accurate individual-level predictions of successful antipsychotic treatment discontinuation, the population-level evidence is clear on the greater risk of relapse and limited advantages associated with treatment interruption, at least in patients fulfilling criteria for schizophrenia. Prescribers should be careful assessing the expressed desire to interrupt treatment, including navigating conflicts of client independence vs medical evidence and ethical duty to not harm. Stakeholder involvement and psychoeducation are critical when making such decisions. In our opinion, unlike what Moncrieff and Horowitz imply, antipsychotic discontinuation after established acute benefits should be the exception rather than the rule in people diagnosed with severe forms of psychotic disorders, such as schizophrenia.
